# Identifying non‐adult attention‐deficit/hyperactivity disorder individuals using a stacked machine learning algorithm using administrative data population registers in a universal healthcare system

**DOI:** 10.1002/jcv2.12193

**Published:** 2023-09-18

**Authors:** David Roche, Toni Mora, Jordi Cid

**Affiliations:** ^1^ Research Institute for Evaluation and Public Policies (IRAPP) Universitat Internacional de Catalunya (UIC) Barcelona Spain; ^2^ Institut d'Assistència Sanitària (IAS) and Mental Health & Addiction Research Group (IDIBGI) Barcelona Spain

**Keywords:** attention‐deficit hyperactive disorder, comorbidity, machine learning

## Abstract

**Background:**

This research project aims to build a Machine Learning algorithm (ML) to predict first‐time ADHD diagnosis, given that it is the most frequent mental disorder for the non‐adult population.

**Methods:**

We used a stacked model combining 4 ML approaches to predict the presence of ADHD. The dataset contains data from population health care administrative registers in Catalonia comprising 1,225,406 non‐adult individuals for 2013–2017, linked to socioeconomic characteristics and dispensed drug consumption. We defined a measure of proper ADHD diagnoses based on medical factors.

**Results:**

We obtained an AUC of 79.6% with the stacked model. Significant variables that explain the ADHD presence are the dispersion across patients' visits to healthcare providers; the number of visits, diagnoses related to other mental disorders and drug consumption; age, and sex.

**Conclusions:**

ML techniques can help predict ADHD early diagnosis using administrative registers. We must continuously investigate the potential use of ADHD early detection strategies and intervention in the health system.

## Graphical abstract

We considered a large‐scale population‐based study applying Machine Learning (ML) to ADHD disease in non‐adults in Catalonia, Spain. The Spanish healthcare system provides free universal healthcare, so we cover all healthcare providers. Our findings show an overall prediction accuracy of more than 84% predicting ADHD diagnosis based on demographic data and health system records. The possible early detection of ADHD is very relevant because of its prevalence of 3.4% of the child‐adolescent population.

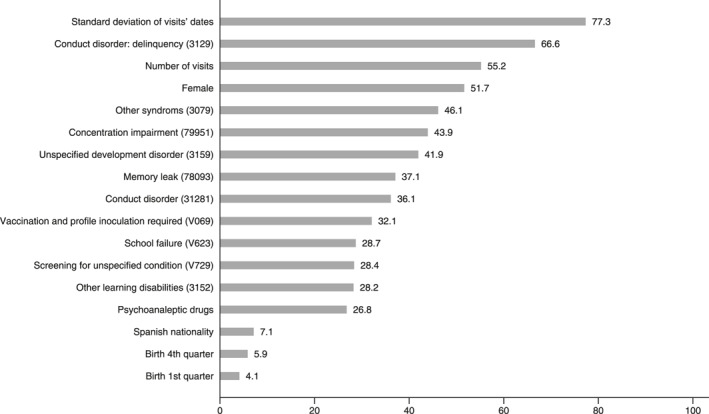

**Key points**

- Predicting ADHD is essential for policymakers in order to anticipate the use of public healthcare resources.
- Unlike most previous studies, we had access to population‐level data, including all kinds of healthcare services: primary care, community, and hospital mental healthcare.
- Early diagnosis should not rely on knowing or detecting early symptoms of ADHD. It is important to consider previous diagnoses recorded as ‘suspicion diagnosis’ in health databases by health professionals to predict ADHD.
- Deploying a predictive model that uses clinical diagnoses in the assessment phase can allow health systems to look at the relevant variables that affect ADHD diagnosis.
- We avoid overdiagnosis by using a restrictive definition of ‘highly likely ADHD’.

## INTRODUCTION

Attention‐Deficit/Hyperactivity Disorder (ADHD) is one of the most prevalent neurobehavioral disorders in children aged 3–17. Reported prevalence rates vary according to the methodologies used to calculate them. A recent USA National Survey of Health (*n* = 26,572) reported prevalence rates for mental disorders, with ADHD at 8.6%. Okwori (2022) estimated prevalence rates of anxiety disorders of 8%. A meta‐analysis study (Polanczyk et al., 2015) found a 3.4% (Confidence Interval 95% 2.6–4.5) ADHD worldwide prevalence in children under 18. In Catalonia, this prevalence was 4.07% in 2017 (Cid & Mora, 2021).

ADHD is characterised by inattention and/or impulsivity and hyperactivity symptoms, which can adversely impact behavioural, emotional, and social aspects of the children's lives. In approximately 80% of children with ADHD, symptoms persist into adolescence and may continue into adulthood (Faraone et al., 2003). The diagnosis is based on observed and reported behavioural symptoms (NICE, 2020). A systematic review by Rocco et al. (2021) showed a high variability in both the age at ADHD diagnosis and the age at onset of the condition. The average age at which children experienced the onset or diagnosis of ADHD was between 2.3 and 7.8 years in the studies they reviewed. However, this age was 15.3 years (with a range from 6.2 to 18.1 years) in children with ADHD and disruptive behaviour. Early diagnosis is critical to implement treatments that might alter the trajectory of the disorders and avoid long‐term negative consequences (Halperin et al., 2012; Sonuga‐Barke & Halperin, 2010).

Predicting Attention‐Deficit/Hyperactivity Disorder (ADHD) first‐time diagnoses through administrative population data is relevant because public administrators can anticipate in advance the use of health care resources and appropriately plan the allocation of professionals to different diseases.

Population‐based administrative databases designed for health system management offer the opportunity to study mental health disorders' epidemiology using longitudinal data (O'Donnell et al., 2022). Electronic health records have been used to demonstrate ADHD diagnosis accuracy (Daley et al., 2014; Mohr‐Jensen et al., 2016). Morkem et al. (2020) reviewed electronic medical records for the diagnosis of ADHD from a primary care database in Canada. The criteria used for a patient to were to be considered an ADHD case, were either whether they had i) equal to or more than one medical visit based on the International Classification of Diseases (ICD‐9), Clinical Modification (ICD‐9‐CM), and equivalent to or more than one prescription of ADHD‐related medications, or ii) more than two medical visits (also based on ICD‐CM code). They found an increase in prevalence between 2008 and 2015. In another study, Gruschow et al. (2016) validated an EHR‐based algorithm that classified ADHD status among a large cohort of primary care patients in a regional healthcare network. Overall, their findings demonstrate that an algorithm that seeks to capture ADHD case status among primary care patients can do so with high sensitivity just using other ICD9‐CM codes for psychiatric conditions contained in visit‐level diagnosis fields or the problem list.

Despite algorithms based on ADHD diagnostic codes from one administrative data source being functional, there is little other evidence. We did not find any research that applied Machine Learning (ML) techniques to diagnose ADHD through database registers. For this reason, we tackle this research question of predicting ADHD non‐adult individuals through ML techniques (ML).

Early detection is one of the critical objectives in mental disorders, especially amongst patients in childhood and adolescence. Screening instruments for mental disorders and ADHD are available based on information from parents and teachers. This helps to ask for and facilitate faster clinical diagnosis, but their use is not widespread in mental health care. Therefore, it is essential to look for other strategies to identify those who seek help because of a suspicion of ADHD and then a follow‐up based on administrative records. In this sense, Sethu and Vyas (2020) presented several ML models to predict ADHD and surveyed previous analyses using ML techniques. Research varies based on the kind of analysed datasets but mainly addresses the problem using cortical features (Biswas et al., 2020; Yasumura et al., 2017), brain circuits (Tang et al., 2019), neuropsychological assessments (Bledsoe et al., 2020), the Continuous Performance Test (Berger et al., 2020; Park et al., 2019) or hyperactivity sensors (Suresh et al., 2020). A recent systematic review highlights how ML can use neurobiological variables and MRI or wearable devices (ECG, PPG and motion data) to develop techniques for ADHD diagnosis (Loh et al., 2022). More recently, Garcia‐Argibay et al. (2023) used registry data to predict ADHD diagnosis. The authors included important variables that predict diagnosis, such as criminal convictions, having a relative with ADHD, or failing subjects at school.

Hence, previous research papers have mainly addressed the question using databases that considered individuals already tested using these various assessments. We depart and contribute from a very different perspective. Specifically, we use previous information that occurred within 2‐year clinical history before the ADHD diagnosis and a balanced control group using populational data and administrative registers in a universal health system. In doing so, we accounted for overdiagnosis through a classification of how potentially likely ADHD diagnoses were. Considering previous organic diagnoses and other mental health diagnoses, medication, the geographical residence that conditions the probability of being diagnosed, and socioeconomic characteristics, we predicted a highly likely ADHD diagnosis with several ML approaches finally combined through a stacked algorithm.

## DATA & METHODS

### Data sources and study population

The Ethical Review Board located in the Hospital Trueta & IAS, Girona (Spain), approved the study. We used a large administrative dataset from The Agency for Health Quality and Assessment of Catalonia (AQuAS) that included information from several providers, considering different time periods for the whole population of Catalan children that were born between 1998 and 2012 (1,225,406 individuals), including those diagnosed with ADHD (49,579) and those not. Note that the Spanish Health System provides universal care that includes mental health care. However, we limited our database. Initially, we used any information that occurred anytime within the previous 2‐year clinical history before first‐time diagnosis, which limited the positive class data to those with 2‐year information before diagnosis. Then, for sensitivity analysis, we limited this clinical history to 1 year and 1 year and a half.

This database contained primary care, hospitalisations, emergency care, mental health hospitalisation, and community mental health care from 2013 to 2017. This was the period requested when submitting the protocol to the regional public administration. In these files we collected the individual identifier, the healthcare provider unit (a total number of 484 healthcare provider units), the date of the visit (length in case of hospitalisations), and all the diagnoses (a total number of 2810 different diagnoses during that period) and procedures (the number of available medical procedures during that period was 463) that were administered. The ICD‐9 diagnostic manual was used in Catalonia until 2017. We considered the specific codes for ADHD: 314, 314.0, 314.00, 314.01, 314.1, 314.2, 314.8, 314.9. Diagnoses were shown in an ordinal sense, indicating the primary diagnosis for that visit and a list of secondary diagnoses. Via unique personal identifiers, the information was linked between all provider's datasets but also to some demographic information: sex, age, drug co‐payment level, which is related to the socioeconomic status of their parents, individual nationality, date of exitus and the sanitary health region they belong to.

### Restricting to proper diagnosis

Measuring potential over‐diagnosis or improper classification of ADHD is essential. We established several definitions of children diagnosed with ADHD: (i) those diagnosed with the ICD‐9 codes related to ADHD in any healthcare provider, (ii) children consuming drugs related to the disease, and (iii) a classification informed by clinical expertise. Specifically, concerning the latter, we categorised children into three groups: (i) highly likely ADHD diagnosis, (ii) potentially likely ADHD diagnosis, and (iii) not very likely ADHD diagnosis. In doing so, we also examined which types of providers diagnosed children with ADHD. Our classification relies on the following rules about how likely it is for ADHD to be present. A highly likely ADHD diagnosis occurs when we find a principal ADHD diagnosis in mental health centres or inpatient mental health units from the comprehensive Catalan public health system (SISCAT). Potentially likely ADHD diagnosis is when we identify an ADHD pharmacological treatment and secondary ADHD diagnosis in mental health centres or inpatient mental health units, or ADHD diagnosis in primary care (we also identified those patients who were diagnosed and treated by private practice, although they might have covered from public pharmacological treatment) from SISCAT. Finally, a not very‐likely ADHD diagnosis occurs when we find the following conditions (a) ADHD pharmacological treatment and ADHD diagnosis in the emergency room or inpatient specialised care (not mental health) from SISCAT, (b) ADHD pharmacological treatment and other mental health diagnoses (no ADHD diagnosis in their clinical record) in mental health centres or inpatient mental health unit from SISCAT, (c) secondary diagnosis without ADHD pharmacological treatment in primary care centres or emergency room or inpatient care (specialised or mental health) or mental health centre from SISCAT.

Then, we restricted our analysis to those individuals classified as highly likely ADHD first‐time diagnosis or potentially likely ADHD diagnosis to avoid overdiagnosis or misdiagnosis issues. Hence, we dropped from the analysis those whose diagnosis was not very likely to be ADHD. Additionally, since the distribution of classes or labels is not uniform due to the excessive population in the negative cases, we randomly undersampled the majority class that is, those who were not diagnosed with ADHD. We randomly selected the 2‐year window once we selected the negative cases using undersampling. After these selection criteria were performed, our database contained 15,890 positives (highly likely ADHD diagnoses) and 15,433 negatives (no ADHD).

### Problem setting

Our outcome corresponds to the event of an ADHD first‐time diagnosis. Given that this outcome is categorical, we are considering a classification task. For this purpose, each patient corresponded to one observation or row in our dataset. For each patient, we had demographic information about sex, socioeconomic level, co‐payment level, nationality, and sanitary health region. Moreover, information on diagnoses and procedures was retained. Finally, ADHD first‐time diagnoses are collected with a dichotomous variable restricted to those highly likely (1 ‐ ADHD, 0 ‐ not ADHD).

### Data pre‐processing

A pre‐processing phase is mandatory because the original data collection was in the event‐row format. For this purpose, we collapsed the dataset at the individual level so that each row contained patient identification, the information registered within medical visits, and sociodemographic variables. First, we created two new variables for each patient: the number of visits during the considered period and the standard deviation of all visit dates. Second, medical visit registers containing diagnoses and procedures carried out during each visit were one‐hot encoded. The same was applied to healthcare provider units. If one variable has *n* categories, *n* new variables were created, assigning one if the patient has that category and 0 if not. For instance, by using binary variables, we identified 2810 medical diagnoses in our database. We excluded the diagnoses related to ADHD from the list of covariates. Then, we identified dummy variables for drug consumption in the Anatomical Therapeutic Chemical classification system. Indeed, we used the one that divided the active substances into three levels (ATC3) according to the chemical, pharmacological or therapeutic subgroup. Next, initially the economic level was a categorical variable with four classes, which was transformed into four dichotomous variables. The same operation was applied to the quarter of birth and drug copayment levels. Nationality was dichotomised to the condition of being Spanish or not. Finally, age was considered continuous. Table 1 summarises how we prepared data after pre‐processing, transformation, and variable creation steps for the tested variables. Data arrangement was carried out using Stata 17.0.

**TABLE 1:** The preparation process of the dataset.

Type	Information	Codification
Event Information	Medical visits	Number of visits
		Date standard deviation
Event Information with One‐Hot encoding	Diagnoses	Dichotomous variable if each diagnosis was made or not
	Procedures	Dichotomous variable if each procedure was made or not
	Health care provider unit	Dichotomous variable if each UP was used or not
	ATC3 drugs	Dichotomous variables representing consumption at ATC3 level
One‐Hot encoding	Sex	Dichotomous variable (female or not)
	Economic‐level	4 level dichotomous variables
	Nationality	Dichotomous variable (Spanish or not)
	Quarter of birth	Dichotomous variables representing quarter of birth
	Copayment	6 level dichotomous variables
Others	Age	Continuous measure

### Machine learning algorithms

Once the data was pre‐processed, a battery of models were estimated. In particular, following the literature, Logistic Regression (LR), Decision Tree (DT), Random Forest (RF), and Extreme Gradient Boosting (XGB) were performed. An 80/20 split of train/test samples was carried out for all models. We standardised all variables for the data transformation stage using z‐score normalisation in the training set. Later, we applied this previous standardisation in the test set. Next, the hyperparameters of each model were tuned using accuracy as a scorer metric. Five‐fold cross‐validation was used in the training set for this hyperparameter setting purpose. Cross‐validation is a known technique to avoid over‐fitting and over‐performed evaluation in ML techniques, dividing the dataset into *k* folds and computing *k* evaluations using one fold for testing and the other to train in each iteration. We considered a feature selection technique to avoid irrelevant or noisy features that can inflate model performance. Specifically, we used the Chi‐square test feature selection technique. This technique calculated the Chi‐square between the feature and the target, selecting the best scores.

Logistic Regression is the most commonly used technique involving binary outcomes for solving classification problems in the medical literature. It calculates the probability of an input belonging to ADHD first‐time diagnosis using a logistic function (a mathematical expression that maps real number values to a value between 0 and 1). In our case, we say that the prediction is positive when it is above 0.5 probability. Among other applications, DT is a classification ML task based on the divide and conquer strategy. The process iterates by repeatedly putting the independent variables into smaller subsets until a stop condition is met. To set which variable is split, Information Gain (IG) measuring the quality of each possibility is computed, and a variable with maximum IG is selected in each iteration. Decision Tree are easy to interpret and can handle both categorical and continuous data. A limitation of DT is that they can suffer from overfitting. Random Forest combines several decision trees, and a restriction over what variables and cases are made is based on hyperparameters setting. The final model classifies instances by majority vote over all tree classifications.

Each tree is built using a random subset of the features and a random sample of the training data. This technique avoids over‐fitting compared to DT and overperforms our metrics, thus better generalising our training data to unseen data. It can be computationally expensive and less interpretable than DT. XGB is an ensemble classification algorithm based on a combination of several weak learners, in this case, DT (a model that can't fit complex data) in a sequential form. It uses a gradient descent algorithm to minimise the regularised objective function, combining the loss function and a penalty term to prevent overfitting. The key point is that each tree deals with cases poorly classified in the previous tree during the process. Often, this scheme outperforms other techniques with better generalisations.

Each algorithm has hyperparameters to control its behaviour. As mentioned above, a cross‐validation technique was performed to tune these hyperparameters for each model with a grid search paradigm. For DT, RF and XGB, a regular grid was computed with five folds. Computations were made using Python 3.9.13. Table 2 indicates the results obtained for the best parameters after the optimisation tuning process.

**TABLE 2:** Hyperparameters for the different machine learning approaches.

Algorithm	Parameter	Grid	Best values
Decision Tree (DT)	min_samples_split	2–100 (5 levels)	2
	max_depth	50–500 (4 levels)	200
Random Forest (RF)	n_estimators	5–100 (4 levels)	50
	max_features	Auto, sqrt (2 levels)	sqrt
	max_depth	10–120 (12 levels)	100
	min_samples_split	2–10 (3 levels)	6
	min_samples_leaf	1–4 (3 levels)	1
	Bootstrap	True, false (2 levels)	false
Extreme Gradient Boosting (XGB)	n_estimators	100 to 2000 (5 levels)	1000
	max_depth	4–40 (5 levels) [0,1]	10
	learning_rate		0.4

*Note*: min_samples_split constitutes a hyperparameter that controls the minimum number of required samples to split an internal node into child nodes, and; max_depth is the maximum number of levels allowed for the trees (longest path from the root node to any leaf node). Both prevent overfitting. *n*_estimators hyperparameter refers to the number of single decision trees created in the forest. max_features hyperparameter controls the maximum number of variables provided to each tree in a RF; min_samples_leaf specifies the minimum number of samples that should be present in any node after splitting it. learning_rate controls the shrinkage of the weights of each tree in the boosting process, that is, controls how much each tree contributes to the final prediction.

Finally, we considered a stacked ensemble method to improve performance. This kind of model generally uses predictions from multiple ML classifiers to produce a better‐predicting model. To some extent, it assigns weights to each model to predict the outcome. Departing from our previous models (LR, DT, RF and XGB), we combined the results using LR as a meta‐learner, which is the most common technique in the literature (Zhou, 2012).

We computed the most common metrics used in ML classification (AUC ‐ Area Under the Curve, Accuracy, Precision, Specificity, Sensitivity and F‐Measure) regarding performance evaluation. The AUC measure uses a receiver operating curve's characteristic to capture the trade‐offs between the actual and false‐positive rates. Values close to one are preferable. Accuracy constitutes the percentage of correctly predicted data from all of the test sets. In contrast, Precision is related to the rate of correctly predicted data within the positive values (presence of the comorbidity). Specificity constitutes the proportion of negative cases correctly classified, whereas Sensitivity refers to the ratio of positive cases correctly identified as positive.

Finally, F‐Measure corresponds to the harmonic mean of Precision and Sensitivity. Since our algorithm outputs a probability indicating the likelihood of the positive class, we chose 0.5 as the threshold to decide if this probability is classified as positive or negative.

A variable importance rank was computed to detect the variables with the highest predicting power in our final stacked model. Variables are important if, after shuffling values, they significantly alter the performance of the model. Our strategy was to compute the importance of variables in each algorithm. For the LR model, we computed these ranks by scaling the standardised coefficients by their maximum value. For the other models (DT, RF and XGB), the variable importance is how much this variable is used in each tree. It was computed using the normalised total reduction of the criterion brought by every variable. We weighted these ranks using the coefficients of each algorithm in the LR applied to obtain the final stacked model.

Finally, we computed the Shapley values to detect the direction of the associations between the predictors and the outcome and to make the models explainable. Shapley values constitute a method from coalitional game theory that tells us how to distribute the pay‐out across the variables fairly. A prediction can be explained by assuming that each variable value of the instance is a *player* in a game where the prediction is the pay‐out. The Shapley value is the average marginal contribution of a variable value across all possible coalitions in that game.

## RESULTS

Table 3 shows descriptive statistics of the randomly selected sample, disentangling by treatment (highly likely ADHD diagnosis or potentially likely ADHD diagnosis and excluding those not very likely to have an ADHD first‐time diagnosis). There are statistically significant differences based on those covariates commonly associated with a higher prevalence of ADHD (the quarter of birth, gender, nationality, and socioeconomic status). However, this significance was affected by the fact that we used a large sample. The only variable not statistically different was age. Figures S1 and S2 in the supplementary material show the most frequent diagnoses and dispensed drugs at the ATC3 level, whereas S3 shows the most frequent procedures. Although it makes an unbalanced sample between the control and the ADHD groups, our initial purpose was to predict rather than search for any causal relationship.

**TABLE 3:** Descriptive statistics of the randomly selected sample (2 years of information).

Variables	ADHD (15,890)	Non‐ADHD (15,435)
Number of visits	14.112 (17.41)	13.315 (15.11)***
Standard deviation of visits dates	139.431 (85.02)	161.472 (83.82)***
Being female	0.261 (0.44)	0.499 (0.50)***
Spanish nationality	0.930 (0.26)	0.873 (0.33)***
Age	12.430 (3.32)	12.474 (4.04)
Exempted copayment	0.064 (0.25)	0.047 (0.21)***
10% copayment	0.084 (0.28)	0.061 (0.24)***
40% copayment	0.565 (0.50)	0.539 (0.50)***
50% copayment	0.277 (0.45)	0.325 (0.47)***
60% copayment	0.007 (0.08)	0.012 (0.11)***
Excluded from copayment	0.003 (0.06)	0.016 (0.13)***
Birth in 1st quarter	0.192 (0.39)	0.248 (0.43)***
Birth in 2nd quarter	0.223 (0.42)	0.242 (0.43)***
Birth in 3rd quarter	0.280 (0.45)	0.256 (0.44)***
Birth in 4th quarter	0.305 (0.46)	0.254 (0.44)***

*Note*: We report mean frequencies and standard deviation in the parenthesis.

*, ** and *** represent statistical significance at a 10%, 5% and 1% levels.

Considering pre‐processed steps and the best hyperparameters obtained from cross‐validation techniques, the results applying each algorithm to the test set are shown in Table 4 (columns 1–4). For this purpose, we used the Chi‐square test as a feature selection technique to choose 120 variables (accuracy saturated at this point). Thus, the remaining variables were associated with the probability of being first‐time diagnosed with ADHD. From the list in Table 1, the following variables remained as covariates: the number of visits, the standard deviation of visits dates, gender, Spanish nationality, all categorical variables proxying the socioeconomic status, the first and the last quarters of birth, one dispensed drug group, two specific procedures, 65 diagnoses and 37 specific healthcare centres.

**TABLE 4:** Performance of predictions through the machine learning approaches for the 2 years.

	Logistic regression	Decision tree	Random forest	Extreme gradient boosting	Stacked (standard error)	Stacked without LR & DT	Stacked in the train set
Accuracy	75.79%	73.20%	78.15%	78.02%	79.63% (0.41%)	78.83%	95.46%
Precision	77.61%	74.06%	79.76%	78.90%	80.15% (0.71%)	78.90%	95.07%
Specificity	78.60%	74.36%	80.44%	79.09%	80.08% (0.81%)	79.44%	94.86%
Sensitivity	73.01%	72.06%	75.89%	76.97%	79.19% (0.91%)	78.24%	96.05%
F‐measure	75.24%	73.05%	77.78%	77.92%	79.67% (0.48%)	78.84%	95.56%
AUC	75.81%	73.21%	78.17%	78.03%	79.64% (0.41%)	78.84%	95.45%

*Note*: We computed standard errors for stacked metrics bootstrapping with 50 replications.

**FIGURE 1:**
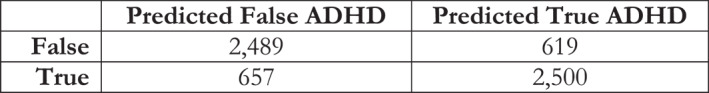
Confusion matrix from the stacked model.

In the final stacked model (Table 4, column 5), all the measures were above 79% general performance. Concretely, accuracy was 79.6%, whereas specificity and sensitivity were 80.1% and 79.2%, respectively. The AUC measure uses a receiver operating curve's characteristic to capture the trade‐offs between the actual and false‐positive rates. Values close to one are preferable. In our case, a value of 79.6% indicated a model of excellent performance, which corroborates the rest of the metrics information. The F‐measure, a metric to compute a trade‐off between precision and sensitivity, was above 79.7%. We show the confusion matrix for the stacked model from which all metrics were computed (Figure 1) and its learning curve (Supplementary Figure S4a). Although it might indicate overfitting, our metrics in the test set still show very good performance (Table 4, column 7). Our values were very high, all around 95%. Given moderate overfitting based on the learning curve (Figure S4a), we computed a stacked model only accounting for RF and XGB (Table 4, column 6) and its learning curve (Supplementary Figure S4b). Our metrics were quite similar but without the presence of this moderate overfitting.

Next, we computed the coefficients of each ML approach for the stacked model. Random Forest was the most contributing technique. The contributions of LR, DT and XGB were 88.8%, 13.4% and 56.1%, respectively, compared to RF.

**FIGURE 2:**
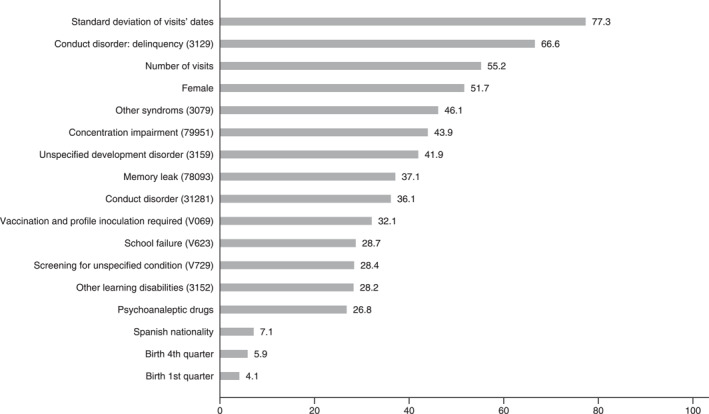
Variable importance rank for the stacked model. Diagnoses include the ICD9 code.

Figure 2 depicts this rank of variables. A larger value (with a maximum of 100) indicates the variables' importance. The different techniques found that the following variables were among the four most important factors to predict the presence of an ADHD first‐.time diagnosis for the non‐adult population: (i) sex (being male the more likely; (ii) some diagnoses such as conduct disorder (including delinquency), concentration impairment, unspecified development disorder, memory leak, school failure and other learning disabilities; (iii) the number of visits and the standard deviation of visits' dates; (iv) first and fourth quarters of birth; and (iv) the condition of being Spanish. To compute the direction of the associations, we calculated the Shapley values (Figure S5 in the supplementary). Our predictions showed that the greater number of visits, the more likely to be diagnosed, whereas the lower the heterogeneity in the visits' dates, the less likely the diagnosis. Those of Spanish nationality were more likely to be diagnosed. A clear pattern was detected for the abovementioned mental diagnoses and dispensed psycho‐analeptic drugs. Thus, the non‐presence of these abovementioned diagnoses was associated with a prediction of non‐ADHD.

**FIGURE 3:**
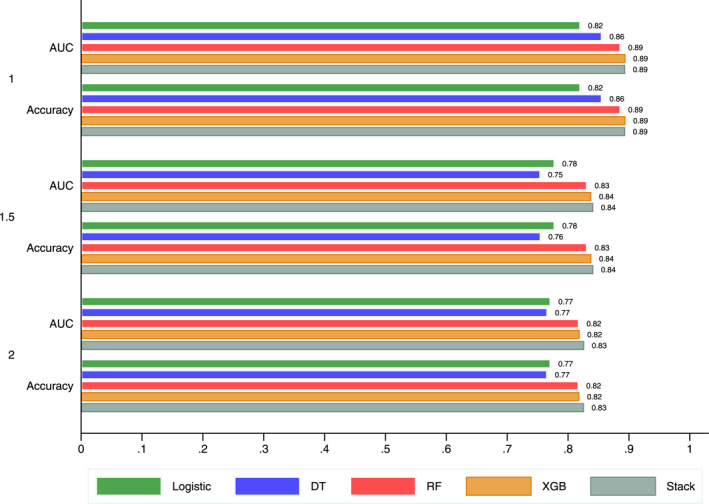
Performance sensitivity analysis to the information period. 1, 1.5 and 2 refer to the considered number of years in each span to account for all the information that occurred before diagnosis.

Then, we tested for sensitivity to the chosen period before the first diagnosis. Indeed, we considered three alternative spans of the clinical history information; anytime within 1 year, 1 year and a half, and within 2 years (which is the baseline value used in our analysis). Figure 3 shows that the more time we consider, the worse the performance of the model. The shorter the period before first‐time diagnosis we consider information for, the better the models perform in predicting ADHD first‐time diagnosis. These findings are specific to our setting (data and ML approaches) and cannot be generalised. In our opinion, it is better to account for the additional information (and diagnoses) when making clinical decisions than to rely on shorter clinical spans.

**FIGURE 4:**
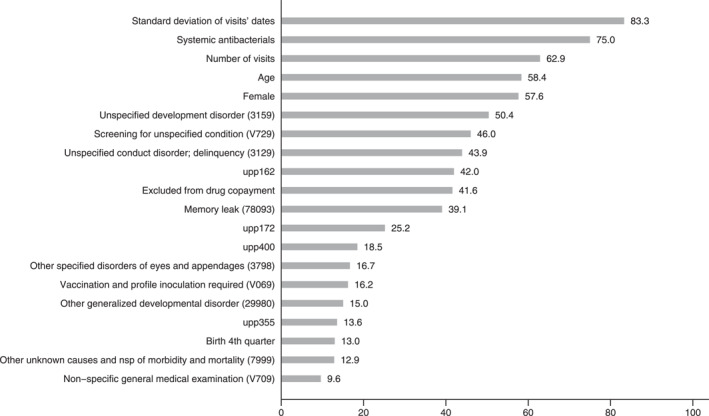
Variable importance rank for the stacked model and matched individuals. Diagnoses include the ICD9 code. *upp* codes referred to specific healthcare centres.

Finally, we consider a model in which we use more comparable individuals instead of the whole dataset to select the random sample information of 2 years. For this purpose, we used neighbourhood matching (considering at least five individuals with the same characteristics: gender, age boundary, asthma comorbidity and the quarter of birth) that was implemented in Mora et al. (2023). This model focuses on finding units that act as a control (the overall population does not constitute a control group per se). Table 5 shows descriptive statistics for these comparable individuals. Even after looking for comparable units through this method, there still appear to be some differences in statistical significance. We ran our ML approaches on this new dataset, obtained similar metrics performance (Table 6), and computed its learning curve (Supplementary Figure S6). Figure 4 shows the variable importance rank results for this approach. Although most previously associated variables with our predictions appeared relevant again (standard deviation of dates' visits, female, number of visits, birth in the 4th quarter, vaccination required), some specific healthcare centres were associated with our predictions using comparable individuals. Besides, fewer mental disorders are associated with these new predictions, although some remain (memory leak, conduct disorder or unspecified development disorder).

**TABLE 5:** Descriptive statistics of the randomly selected sample (2 years of information) and matched individuals.

Variables	ADHD (915)	Non‐ADHD (915)
Number of visits	12.019 (13.74)	12.743 (16.48)
Standard deviation of visits dates	137.379 (89.11)	163.247 (87.04)***
Being female	0.250 (0.43)	0.423 (0.49)***
Spanish nationality	0.966 (0.18)	0.914 (0.28)***
Age	12.692 (2.78)	13.174 (3.42)***
Exempted copayment	0.062 (0.24)	0.054 (0.23)
10% copayment	0.077 (0.27)	0.056 (0.23)*
40% copayment	0.506 (0.50)	0.513 (0.50)
50% copayment	0.336 (0.47)	0.339 (0.47)
60% copayment	0.017 (0.13)	0.020 (0.14)
Excluded from copayment	0.002 (0.05)	0.020 (0.14)***
Birth in 1^st^ quarter	0.188 (0.39)	0.226 (0.42)**
Birth in 2^nd^ quarter	0.219 (0.41)	0.255 (0.44)*
Birth in 3^rd^ quarter	0.272 (0.45)	0.278 (0.45)
Birth in 4^th^ quarter	0.321 (0.47)	0.242 (0.43)***

*Note*: We report mean frequencies and standard deviation in the parenthesis.

*, ** and *** represent statistical significance at a 10%, 5% and 1% levels.

**TABLE 6:** Performance of predictions through the machine learning approaches for the 2 years and matched individuals.

	Logistic regression	Decision tree	Random forest	Extreme gradient boosting	Stacked (standard error)	Stacked in the train set
Accuracy	78.33%	72.23%	77.20%	73.81%	80.59% (1.61%)	90.34%
Precision	78.34%	72.02%	76.58%	73.30%	80.37% (2.54%)	89.69%
Specificity	79.02%	72.77%	76.79%	73.66%	80.80% (2.93%)	89.27%
Sensitivity	77.63%	71.69%	77.63%	73.97%	80.37% (2.62%)	91.40%
F‐measure	77.98%	71.85%	77.10%	73.64%	80.37% (1.77%)	90.54%
AUC	78.32%	72.23%	77.21%	73.82%	80.58% (1.60%)	90.33%

## DISCUSSION

A stacked model based on 4 ML prediction techniques was applied to predict the occurrence of short‐term ADHD first‐time diagnoses based on two previous years' information collected from an extensive population database containing information related to demographic, socioeconomic, diagnoses, medication, and procedures. Indeed, an interesting point of our study was collecting information relating to the population of highly likely ADHD diagnoses instead of a sample. This increases confidence for our predictions. The strategy's framework selected a window of two previous years homogeneously for positive and negative ADHD diagnoses. To avoid overdiagnoses, we chose an indicator for those highly likely to be diagnosed with ADHD.

A battery of many convenient metrics for binary prediction was used to assess the algorithms' performance. Then, a stacked model strategy was implemented to create a combined final model that outperforms all of the individually defined models. Random Forest was the highest weighted among the complementary algorithms considered in the stacked model. Using a populational dataset from administrative healthcare use registers of Catalonia's non‐adult population, we can predict ADHD first‐time diagnosis reasonably well, with a maximum AUC of 0.80.

It is useful to check the most important variables in the model to understand how the mental health system should be organised to diagnose and treat mental health problems, including ADHD. One of the more important variables is the dispersion across visit dates; our outcome showed that less dispersion (probably because symptoms were less obvious) indicates a lower likelihood of having an ADHD diagnosis. This is evidence to support the early intervention of the mental health system (NICE, 2020).

Furthermore, data indicated no standard procedure for ADHD diagnosis in child‐adolescent mental health centres. The Catalan Health System establishes types of visits in severity (urgent or ordinary), and centres can organise the ADHD assistance process.

An interesting additional variable in our models is the number of visits before ADHD diagnosis. This can be explained by how the Catalan Health system is organised. We have a gate‐keeping system from primary care to specialised mental health centres. Generally, first contact for those with a health problem, including mental health, is in primary care. When paediatrics or primary doctors visit the patient, it is mandatory under law to register the health trouble in an electronic clinical record for each contact. ADHD diagnosis is not recorded/labelled in this contact before diagnosis and differential diagnosis. It is difficult to detect multiple neurocognitive and behavioural alterations involved in the early development of ADHD (Shephard et al., 2021). If the patient is referred to a specialised mental health centre, the clinical datasheet can include alterations or unspecific symptoms that can be understood as suspicion of ADHD. When mental health professionals, psychiatrists or clinical psychologists visit the patient and begin the assessment and diagnostic process, they must enter a suspected diagnosis in the electronic clinical record. That is why we find different diagnostics in the health system before the ADHD diagnostic. Then, the results related to other mental diagnoses are significant because they indicate the trend of diagnoses used by mental health professionals when identifying ADHD. This predictive capacity of a diagnosis of ADHD based on other previous (suspected) diagnoses is important. This is because when a person is seen in a child and youth mental health centre for the first time, it is mandatory to indicate an initial suspected diagnosis. The diagnosis will be modified in subsequent visits after the diagnostic evaluation is completed, which normally takes between two and four visits. This will then be considered the main mental disorder diagnosis. This set of previous diagnoses can help the clinician make a faster diagnosis and facilitate early treatment. The same reasoning can be used when looking at pharmacological prescriptions. Before finding ADHD medication in the patients' records, we can find other medication uses in the function of symptoms or alterations observed and the impact on the life of a child or adolescent.

Finally, age and sex are variables studied in diagnosing ADHD; these variables have an essential role, and more ADHD diagnoses occur in male adolescents, as shown in previous epidemiological studies (Thomas et al., 2015; Willcutt, 2012; Xu et al., 2018).

Some limitations are worthy of mention. First, we used the whole Catalan population consuming public healthcare resources. The latter would guarantee external validation, but we do not have access to the population using private healthcare resources. Although one strength was the use of public healthcare administrative records, recent literature has used registry data that include factors associated with educational or judicial backgrounds (Garcia‐Argibay et al., 2023).

In conclusion, we have presented evidence showing that we can predict ADHD diagnosis through ML approaches using administrative data population registers. There is the first study applied to ADHD diagnosis and it is important to replicate these methods using data from other health systems.

## AUTHOR CONTRIBUTIONS

**David Roche**: Conceptualization; data curation; formal analysis; methodology; software; writing—original draft. **Toni Mora**: Conceptualization; data curation; formal analysis; funding acquisition; methodology; software; supervision; visualization; writing—original draft; writing—review and editing. **Jordi Cid**: Investigation; resources; validation; writing—original draft; writing—review and editing.

## CONFLICT OF INTEREST STATEMENT

An unrestricted grant from the Ministry of Science and Innovation from the Government of Spain supported this research. The authors have declared that they have no competing or potential conflicts of interest.

## ETHICAL CONSIDERATIONS

The Ethical Review Board located in the Hospital Trueta & IAS, Girona (Spain), approved the study.

## Supporting information

Supporting Information S1

## Data Availability

The data sets analysed during the current study are private because they consist of data from administrative registers that belong to the regional public administration and, therefore cannot be shared. Jordi Cid and Toni Mora obtained permission to use this data from public administration.
