# Supplementary material for: Identifying non‐adult attention‐deficit/hyperactivity disorder individuals using a stacked machine learning algorithm using administrative data population registers in a universal healthcare system
Source: JCPP Adv. 2023 Sep 18;4(1):e12193. doi: 10.1002/jcv2.12193 (PMC10933630; doi:10.1002/jcv2.12193)
Supplement: Supplementary file 1 — Supporting Information S1 [file JCV2-4-e12193-s001.docx]

Supplementary

Figure S1 Most frequent diagnoses for the randomly selected sample.

|  |
| --- |

Figure S2. Most frequent dispensed drugs at the ATC3 level for the randomly selected sample.

|  |
| --- |

Figure S3. Most frequent medical procedures for the randomly selected sample.

|  |
| --- |

Figure S4a. Learning curves for the stacked model accounting for all algorithms.

| 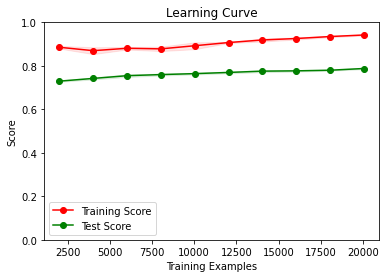 |
| --- |

Figure S4b. Learning curves for the stacked model without LR & DT.

| 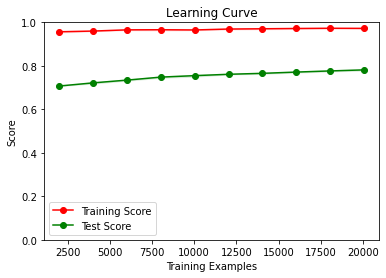 |
| --- |

Figure S5. Shapley values for a sample of 500 individuals.

| 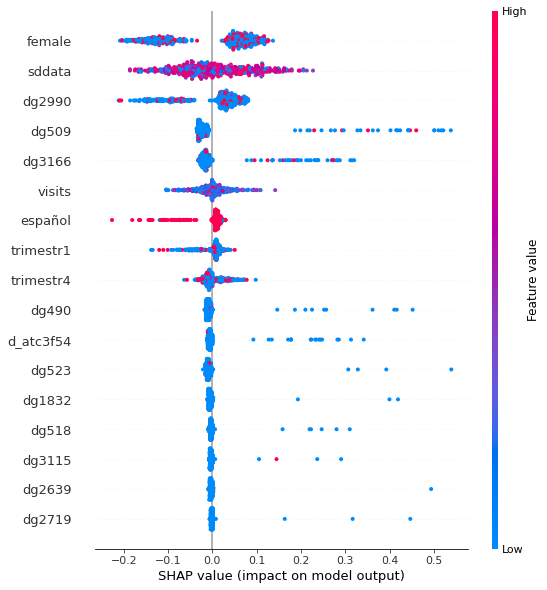 |
| --- |

Note: labelled diagnoses are shown in Figure 2. *d_atc3f54* refers to dispensed psycho-analeptic drugs. *español* indicates Spanish nationality; *visits* represents the number of visits, and *sddata* is the standard deviation of visits’ dates. *trimestr1* and *trimestr4* corresponds to the first and fourth quarter of birth.

Figure S6. Learning curves for the stacked model and matched individuals.

| 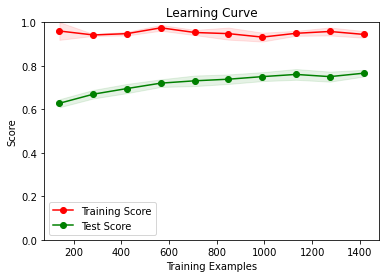 |
| --- |
